# Dysfunctional Coping Mechanisms Contribute to Dry Eye Symptoms

**DOI:** 10.3390/jcm8060901

**Published:** 2019-06-24

**Authors:** Sneh Patel, Elizabeth R Felix, Roy C Levitt, Constantine D. Sarantopoulos, Anat Galor

**Affiliations:** 1 Ophthalmology and Research Services, Miami Veterans Affairs (VA) Medical Center, Miami, FL 33125, USA; sbpatel@med.miami.edu (S.P.); efelix@med.miami.edu (E.R.F.); 2 Bascom Palmer Eye Institute, University of Miami, Miami, FL 33136, USA; 3 Department of Physical Medicine & Rehabilitation, University of Miami, Miami, FL 33136, USA; 4 Department of Anesthesiology, Perioperative Medicine and Pain Management, University of Miami, Miami, FL 33136, USA; rlevitt@med.miami.edu (R.C.L.); ksarantopoulos@med.miami.edu (C.D.S.)

**Keywords:** dry eye disease, coping, catastrophizing, chronic pain

## Abstract

Dysfunctional coping behaviors, such as catastrophizing, have been implicated in pain severity and chronicity across several pain disorders. However, the impact of dysfunctional coping has not been examined under the context of dry eye (DE). This study evaluates relationships between catastrophizing and measures of DE, including pain severity and pain-related daily interference. The population consisted of patients seen at Miami Veterans Affairs eye clinic between April 2016 and October 2017. Patients filled out standardized questionnaires assessing symptoms of DE and eye pain, non-ocular pain, mental health, coping behaviors (Pain Catastrophizing Scale, PCS), and pain-related daily interference as a perceived impact on quality of life (Multidimensional Pain Inventory, Interference Subscale, MPI-Interference), and all patients underwent an ocular surface examination. In total, 194 patients participated, with a mean age of 58.8 ± 9.6 years, the majority being male, non-Hispanic, and black. PCS (catastrophizing) was correlated with DE symptom severity, including Dry-Eye Questionnaire 5 (DEQ5; r = 0.41, *p* < 0.0005), Ocular Surface Disease Index (OSDI; r = 0.40, *p* < 0.0005), and neuropathic-like eye pain (Neuropathic Pain Symptom Inventory-Eye (NPSI-Eye; r = 0.48, *p* < 0.0005). Most tear metrics, on the other hand, did not correlate with PCS. Linear regressions showed that PCS, non-ocular pain intensity, and number of pain conditions were significant predictors of DEQ5 (overall DE symptoms), while PCS and non-ocular pain intensity were predictors of NPSI-Eye scores, as were insomnia scores and analgesic use. In a separate analysis, PCS and DE symptoms (OSDI) associated with pain-related interference (MPI-Interference) along with non-ocular pain intensity, post-traumatic stress disorder (PTSD), number of pain conditions, and non-Hispanic ethnicity. These findings suggest that catastrophizing is not significantly related to signs of DE, but is strongly associated to pain-related symptoms of DE and daily interference due to pain.

## 1. Introduction

Dry eye (DE) is a common, multifactorial disease that can result from abnormal tear film, ocular surface, and somatosensory pathways. DE symptoms are a source of significant morbidity, causing chronic sensations of pain, described in terms of “dryness”, “burning”, and “irritation” (herein referred to as pain-related DE symptoms) and fluctuations or loss of vision (vision-related symptoms). Together, these symptoms can negatively impact physical and mental functioning [1,2]. In this regard, DE has much in common with chronic pain disorders outside the eye, particularly with chronic neuropathic pain conditions [3].

Various factors can negatively impact the pain experience, including older age, female gender, low socioeconomic status, and smoking and alcohol abuse [4,5]. Similarly, psychosocial factors can also affect the pain experience, as anxiety, depression, inadequate social support, and perceived lack of control over pain have all been associated with increased pain levels [6,7,8]. Similar factors have been implicated in the report of DE, as anxiety and depression both significantly correlated with DE diagnosis and symptom severity [9,10,11].

Coping strategies can be defined as the organizational behavioral constructs used to encompass the myriad actions individuals use to deal with stressful experiences [12]. Coping strategies, as thoughts and actions used by patients to deal with or avoid pain, constitute another psychosocial factor that can influence perception and manifestations of pain. Active coping strategies, characterized by reliance on oneself to function in spite of pain (e.g., task persistence or positive self-statements when in pain), are considered healthy, whereas passive strategies that allow daily life to be changed by pain (e.g., relying on external aid during painful tasks, pain avoidance, and catastrophizing) can lead to a sense of helplessness and are considered dysfunctional [13,14]. Studies have shown that patients who employ active coping display better adjustment to pain, showing significantly lower signs of pain severity, disability, and depression than those who rely upon passive strategies [15,16].

Catastrophizing can be broken down into three underlying psychological mechanisms: (1) rumination or overthinking (e.g., “I can’t stop thinking about how much it hurts”), (2) magnification (e.g., “I´m afraid something serious might happen”), and (3) helplessness (e.g., “There is nothing I can do to reduce the severity of my pain”). All three have been found to negatively impact the pain experience [17]. In patients with musculoskeletal pain, catastrophizing mildly correlated with pain severity (r = 0.30, *p* < 0.01) and moderately with interference on quality of life (r = 0.43, *p* < 0.01) as well as poor mental health (r = 0.54, *p* < 0.01) [18]. When analyzing a list of common coping strategies, catastrophizing was the strongest predictor of poor mental health (r = −0.58, *p* < 0.0026) and second strongest predictor of daily interference (r = 0.67, *p* < 0.0026) in a population of 157 patients with pain secondary to spinal cord injury. This highlights the need to address catastrophizing as an important behavioral factor when providing treatment for chronic pain [16].

One instrument used to assess catastrophizing is the Pain Catastrophizing Scale (PCS) [19]. This survey rates the degree to which patients experience catastrophic thoughts of rumination, magnification, and helplessness on a scale of 0 (never) to 4 (at all times), with a score of 30 indicating a clinically relevant level. As discussed above, studies have examined the effects of catastrophizing in patients with non-ocular pain conditions through use of the PCS and other instruments [19,20,21,22]. But, this relationship has yet to be explored for pain-related DE symptoms. Recent studies have suggested similarities between DE symptoms in some patients and non-ocular pain conditions, giving rise to the potential for catastrophizing to promote the negative impact DE has on life [23]. Based on this rationale, the current study investigated the relationship between catastrophizing and various measures of DE, testing our hypothesis that reliance on catastrophizing can have a negative impact on pain-related DE symptom severity as well as on daily quality of life (QoL) via heightened pain-related interference.

## 2. Methods

The study’s Institutional Review Board (IRB) protocol listed as follows—Miami VA IRB 3011.04.

### 2.1. Study Population

Patients with normal eyelid and corneal anatomy were prospectively recruited from the Miami Veterans Affairs (VA) Healthcare System eye clinic between April 2017 and October 2017. For this study, every patient underwent a complete ocular surface exam. Subjects were excluded if they had a condition which could confound DE, including: contact lens use, history of refractive surgery, ocular medications with exception of artificial tears, an active external ocular process, cataract surgery within the last six months, any glaucoma or retinal surgery, human immunodeficiency virus, sarcoidosis, graft-versus host disease, or Sjögrens. Conjunctivochalasis, eyelid laxity, and diabetes were not part of the exclusion criteria given their high prevalence in this population. Informed consent was obtained from all subjects. Miami VA Institution Review Board approval was obtained to allow the prospective evaluation of subjects. The study was conducted in accordance with the principles of the Declaration of Helsinki and complied with the requirements of the United States Health Insurance Portability and Accountability Act.

The study population consisted of 194 individuals who were seen at the Miami VA eye clinic. The Miami VA eye clinic serves a diverse population of patients including those with refractive errors, medical conditions that require monitoring (e.g., diabetes), and ocular conditions. Patients seen in the clinic were offered recruitment into the study if they met inclusion and exclusion criteria, which included individuals with and without dry eye.

### 2.2. Measures

For each subject, demographics (age, sex, race, ethnicity), past ocular and medical history, and medication information was collected. 

*Dry eye symptoms*: Patients filled out standardized questionnaires regarding dry eye symptoms, including the Dry Eye Questionnaire 5 (DEQ5; score 0–22) [24] and the Ocular Surface Disease Index (OSDI; score 0–100) [25].

*Ocular pain*: Pain questionnaires were used to assess the severity and quality of ocular pain. A numerical rating scale (NRS; score 0–10) was used to assess the “average intensity of eye pain during the past week”. The Neuropathic Pain Symptom Inventory (NPSI) [26], modified for the eye (NPSI-Eye) [27], was used to quantify the severity of different dimensions of neuropathic pain. In order to modify the original NPSI so that it was relevant to ocular pain, we replaced three of the original questions regarding the severity of allodynia or hyperalgesia caused by (1) light touch, (2) pressure, or (3) contact with something cold on the skin, with questions specific to *ocular* allodynia or hyperalgesia (eye pain caused or increased by (1) wind, (2) light, and (3) heat or cold). Each question was rated on a scale of 0–10, resulting in a total score between 0 and 100.

*Ocular surface and corneal sensitivity evaluation*: All patients underwent a standardized examination that included, in the order performed (1) tear osmolarity (TearLAB, San Diego, CA, USA), once in each eye; (2) mechanical detection and pain thresholds of the right central cornea assessed with a modified Belmonte non-contact aesthesiometer [28], (3) ocular inflammation assessed with InflammaDry (Quidel, San Diego, CA, USA), once in each eye and qualitatively graded on a scale of 0 (none) to 3 (severe) based on the intensity of the pink stripe; (4) tear evaporation measured via tear breakup time (TBUT) (5 µL fluorescein placed, 3 measurements taken in each eye and averaged); (5) corneal epithelial cell disruption measured via corneal staining (National Eye Institute (NEI) scale, 5 areas of cornea assessed; score 0–3 in each, total 15); (6) tear production measured via Schirmer’s strips with anesthesia; and (7) meibomian gland assessment. Eyelid vascularity was graded from 0 to 3 (0 none; 1 mild engorgement; 2 moderate engorgement; 3 severe engorgement) and meibum quality from 0 to 4 (0 = clear; 1 = cloudy; 2 = granular; 3 = toothpaste; 4 = no meibum extracted).

*Non-ocular pain:* Pain questionnaires were used to assess the intensity and type of non-ocular pain. A numerical rating scale (NRS; score 0–10) was used to assess “average intensity of pain in the body (other than eye) in the past week”. Subjects were also asked to indicate presence or absence of chronic pain conditions (≥3 months) from a list of 21 total, including headache, fibromyalgia, post-herpetic neuralgia, cancer pain, etc.

*Mental health and insomnia*: Symptoms of post-traumatic stress disorder (PTSD) were assessed via the PTSD Checklist—Military Version (PCL-M), score range 17–85 [29,30]. Symptoms of depression were assessed via the patient health questionnaire 9 (PHQ9), score range 0–27 [31]. Insomnia severity was assessed via the Insomnia Severity Index (ISI), score range 0–12 [32].

*Catastrophizing*: The Pain Catastrophizing Scale (PCS) [19] was used to quantify dysfunctional coping behaviors. The PCS score is calculated as a sum of three subscales (rumination, magnification, and helplessness) with a total range of 0–52.

*Pain-Related Interference:* The Multidimensional Pain Inventory—Interference Subscale (MPI-Interference) [33] was used to quantify the impact of eye pain on daily functions. The MPI-Interference score is calculated on a range of 0–7, with a higher score indicating greater interference of pain with daily activities and social functioning.

### 2.3. Statistical Analysis

Analyses were performed using SPSS 22.0 [34]. Descriptive statistics were used to summarize demographic and clinical information. Distribution normality was confirmed using the Kolmogorov-Smirnov (K-S) test for each outcome variable. Pearson correlations were used to evaluate strength of association between pain catastrophizing (PCS) and DE measures, while Student’s t-tests were used to evaluate differences in means across groups. Linear regressions with forward selection were used to evaluate the contribution of variables to the variability measured in DE symptoms. We inspected residuals from linear regression for departures from normality and heterogeneity. In this paper, we opted to give information on all variables being compared as opposed to correcting the *p*-value (e.g., Bonferroni), since the latter methodology has its own limitations [35].

## 3. Results

*Study population and demographics*: The study population consisted of 194 individuals seeking care at the Miami VA eye clinic. The mean age was 58.8 ± 9.6 and the majority of patients were male (89.0% of patients), black (63.0%), and non-Hispanic (78.9%) (Table 1). Several co-morbidities were present in the group, including diabetes (31.4%), osteoarthritis (53.1%), sleep apnea (24.7%), benign prostatic hypertrophy (16.0%), hypertension (60.3%), and hypercholesterolemia (50.0%).

*Relationship between demographics with catastrophizing and pain interference*: The mean PCS score was 18.6 ± 15.8, range 0–52, and the mean MPI-Interference score was 2.2 ± 1.9, range 0–6. The correlative relationship between PCS (catastrophizing) and MPI-Interference (pain-related interference) was moderately strong in magnitude (r = 0.62, *p* < 0.0005). Demographic measures (e.g., gender, race, and ethnicity) and the studied co-morbidities (e.g., smoking, diabetes mellitus, osteoarthritis, sleep apnea, benign prostatic hyperplasia, hypertension, and hypercholesterolemia) were not associated with PCS scores. However, subjects on anti-depressants (mean 22.7 ± 16.3 vs 13.1 ± 13.3, *p* < 0.0005), anxiolytics (23.0 ± 16.5 vs 12.9 ± 12.9, *p* < 0.0005), analgesics (21.4 ± 16.4 vs 13.9 ± 13.7, *p* = 0.001), and anti-histamines (25.4 ± 15.8 vs 16.6 ± 15.3, *p* = 0.001) showed higher PCS scores than individuals not using these medications. For MPI-Interference, gender differed significantly, with female subjects on average having greater pain interference than male subjects (3.1 ± 1.8 vs 2.2 ± 1.8, *p* = 0.03). Patients with osteoarthritis reported lower pain interference than subjects without the condition (2.5 ± 1.9 vs 1.9 ± 1.9, *p* = 0.02). As with the PCS, subjects on anti-depressants (2.7 ± 1.9 vs 1.6 ± 1.7, *p* < 0.0005), anxiolytics (2.8 ± 1.9 vs 1.5 ± 1.7, *p* < 0.0005), and analgesics (2.6 ± 1.9 vs 1.7 ± 1.8, *p* = 0.001) had higher pain interference with daily life compared to their counterparts who did not use these medications.

*Relationship between DE and pain catastrophizing*: All DE symptom measures, including eye pain were moderately correlated with PCS score, demonstrated by Pearson correlation coefficients of approximately 0.45. (Table 1) Among all ocular surface measures, only Belmonte sensory detection threshold and ocular surface inflammation measured by InflammaDry correlated with PCS score, both with a negative relationship at a Pearson coefficient just over −0.15. Non-ocular pain indices (e.g., non-ocular pain intensity and number of pain conditions) and other co-morbidities related to chronic pain (depression, PTSD, insomnia) were all positively correlated with PCS score at a moderate magnitude.

Factors that influence pain-related daily interference (MPI-Interference): A stepwise linear regression model was used to understand how DE symptoms, co-morbidities, and catastrophizing (PCS) related to pain interference (MPI-Interference). Using the same independent variables as above, catastrophizing score (PCS), non-ocular pain intensity of the past week, PTSD score (PCL-M), number of chronic pain conditions, non-Hispanic ethnicity, and OSDI remained associated with MPI-Interference, explaining 58% of variability (R = 0.76) (Table 2).

Impact of catastrophizing on DE symptoms (overall and pain-related) while considering co-morbidities: Stepwise linear regressions were also used to evaluate associations between catastrophizing and DE symptom severity. Considering the demographics, ocular parameters, and co-morbidities that were significantly correlated in regression models, we found that average non-ocular pain intensity, number of pain conditions, and catastrophizing (PCS) explained 30% of variability in DEQ5 (R = 0.55). Comparable findings were seen for a model involving OSDI—Non-ocular pain intensity, depression (PHQ9), and catastrophizing (PCS) explained 37% of variability (R = 0.61). When analyzing pain-related symptoms, variables that associated with ocular pain intensity included non-ocular pain intensity, depression (PHQ9), and catastrophizing (PCS) (38% variability, R = 0.63) while variables that associated with NPSI-Eye scores were non-ocular pain intensity, insomnia, catastrophizing (PCS), and analgesic use (43% variability, R = 0.66). Of interest, inclusion of PCS increased the overall R-value and percent variability in the outcome variable for every model. Overall, these analyses demonstrate that catastrophizing was a significant predictor for every DE symptom measure (overall and pain-related).

## 4. Discussion

Results from this study support a strong association between the use of catastrophic thinking as a coping strategy for pain and both the report of eye pain intensity as well as its interference on daily life. DE symptom severity and catastrophizing related to pain-related daily interference, both in isolation and in combination. Prior studies have found similar negative effects of DE on QoL. A study using the Impact of Dry Eye on Everyday Life questionnaire found that DE symptoms associated with difficulties in physical, social, and mental functioning [36]. Symptoms have been found to impact various domains, including reading, professional work, computer use, television watching, and driving [37], and severe symptoms have even been linked to depression and suicidal ideation [38]. Likewise, a link between catastrophizing and functional interference (daily activities, mood, relationships, etc.) has been previously reported. In 62 adult patients with a variety of chronic pain conditions, PCS was associated with interference measured by MPI-Interference (r = 0.38, *p* < 0.05) [39], as it was in 214 patients with temporomandibular joint disorder (r = 0.52, *p* < 0.05) as measured by BPI (Brief Pain Inventory) [40]. Of note, this connection has been reproduced in the laboratory setting—in a study of 48 healthy adults, subjects with higher PCS recorded increased pain interference, measured as a change in reaction time from baseline (r = 0.33, *p* = 0.024), during attention-demanding auditory discrimination tasks while receiving electrocutaneous stimulation [41].

Eye pain is often part of the symptom profile associated with DE, and, indeed DE symptom severity (both overall and pain-related) significantly associated with pain catastrophizing. These associations remained even after controlling for co-morbidities that can affect pain, including anxiety, depression, and insomnia. The relationship was moderate in strength (r = ~0.45), which coincides with studies relating catastrophizing to pain in disorders outside the eye. In a population of 40 adults with chronic low back pain, a Pearson correlation of r = 0.39 was noted between catastrophizing (PCS) and current pain intensity (McGill Pain Questionnaire, range 1–5) [42]. Similar relationships between catastrophizing and current pain were observed in 48 adults with pain after surgical anterior cruciate ligament repair (r = 0.48, *p* = 0.004, between PCS and 11-point Likert scale), and in 64 individuals with postoperative cardiothoracic pain (r = 0.41, *p* < 0.0005, between PCS and 10-point Verbal Rating Scale) [43,44]. Our findings further suggest that catastrophizing associates with higher pain intensity in patients with ocular pain as it has been found to do in multiple pain conditions outside the eye.

Interestingly, catastrophizing did not associate with most pathologic ocular surface signs, except for corneal sensitivity and ocular surface inflammation. Patients with higher PCS were more sensitive to a puff of air to the central cornea and had less inflammation of the ocular surface. Considering corneal sensitivity, hyperesthesia to stimuli has been linked to catastrophizing in other pain states [45,46]. A study on 24 upper extremity amputees reported a negative relationship between PCS and sensation proximal to the stump for cold (β = −0.295; R^2^ = 0.506; *p* = 0.006) and mechanical-tactile (β = 0.083; R^2^ = 0.463; *p* = 0.012) thresholds (for mechanical, a strong stimulus was presented and stimulus strength was progressively lessened, thus a positive β indicates that catastrophizing associated with higher sensitivity) [47]. Similarly, in 25 women with vulvar vestibulodynia, lower heat (r = −0.45, *p* < 0.05) and heat-pain (r = −0.48, *p* < 0.05) detection thresholds related to higher PCS [48]. The finding of an inverse relationship between catastrophizing and inflammation is not as clear, but higher intensity of painful symptoms in the absence of inflammation may be driven by neuropathic mechanisms that are disconnected from ocular signs [49]. For these cases, central mechanisms may drive pain-related symptoms, and the underlying centralized disorder(s) may contribute to mental, emotional and behavioral processes related to catastrophizing, as well [50].

There is biologic plausibility for a connection between catastrophizing and DE symptom severity. Three mechanisms have been described by which catastrophizing may impact pain sensitivity pathways and increase pain perception [51]. First, catastrophizing may induce alterations in molecular processes that can affect pain, such as by causing sustained diurnal cortisol levels throughout the day, or heightening the pro-inflammatory cytokine (IL6) response to noxious stimuli in systemic circulation, which may further enhance cognitive processes that maintain catastrophizing [52,53]. Next, catastrophizing may impede the central nervous system’s ability to modulate incoming pain signals by preventing temporal summation through central sensitization-like processes (preventing modulation during repetitive painful stimuli) and by inhibiting diffuse noxious inhibitory controls that attenuate activated dorsal horn neurons (preventing endogenous inhibition of incoming pain signals), leading to heightened pain perception [54,55]. Finally, brain imaging has shown that catastrophizing may alter pain perception and affective responses to pain by promoting aberrant activity in brain cortices involved with pain anticipation (medial frontal cortex and cerebellum), attention (dorsal anterior cingulate cortex and prefrontal cortex), and emotional responses to pain (claustrum) [56,57,58]. It is possible that these central changes in total may impact perceived pain-related DE symptoms as well.

As with all studies, our findings must be considered in light of the study’s limitations, which included a specific population that limits generalizability of our results. Additionally, specific self-reported questionnaires were used to assess catastrophizing and pain-related daily interference. Furthermore, there is potential for unmeasured confounders to impact our data, including sub-clinical allergic disease, which may confound DE symptoms [59,60]. Finally, while we found a correlation between catastrophizing and DE symptoms, our study methodology was not designed to establish causality. Therefore, possibilities include that dysfunctional coping leads to chronic DE symptoms, that the presence of chronic DE symptoms may help establish dysfunctional coping behaviors, or that underlying confounders drive both manifestations. Further studies are needed to assess the directionality of these noted associations. Despite these limitations, our results suggest that catastrophizing is associated with DE symptom severity and interference with daily life, but not with DE signs. This supports prior research that DE symptoms may be more closely related to altered central processes than to pathological tear film changes [10,23,61,62].

## 5. Conclusions

Our findings have implications with regards to treatment of DE. The majority of current therapies focus on improving ocular surface pathologies to alleviate symptoms, while our results indicate a potential utility in targeting behavioral (e.g., coping-related) and systemic findings. One study on low back pain found that intervening on patients who catastrophize via cognitive behavioral therapy (problem solving therapy such as redefining the problem and graded activity techniques such as focusing on other goals) or active physical treatment (aerobic training and strength-endurance training) lessened reliance on catastrophizing and led to less impact of pain on QoL [63]. Our findings indicate that similar strategies could help reduce morbidity in patients with DE symptoms after targeting pathological ocular findings. Therefore, additional studies exploring the potential of interventions pertinent to coping with chronic pain should be considered. These findings highlight that, as with chronic non-ocular pain, comprehensive and multidisciplinary patient centered-approaches delivered by teams of expert clinicians may be necessary to achieve optimal therapeutic outcomes in patients with DE.

## Figures and Tables

**Table 1:** Clinical Characteristics of the Population and Relationships to Catastrophizing (PCS) and Pain-Related Interference (MPI-Interference).

Demographics	Mean (SD)	PCS (r)	*p*-Value	MPI (r)	*p*-Value
Age (years)	58.8 (9.6)	−0.11	0.14	−0.17	0.02
**Medication Use**	***n* (%)**				
Antidepressant	111 (57.2%)				
Anxiolytic	110 (56.7%)				
Analgesic	123 (63.4%)				
Antihistamine	45 (23.2%)				
**DE symptoms (Overall)**	**Mean (SD)**				
DEQ5 (0–22)	11.2 (5.2)	0.41	<0.0005	0.40	<0.0005
OSDI (0–100)	37.2 (23.8)	0.49	<0.0005	0.54	<0.0005
**DE symptoms (Pain-related)**	**Mean (SD)**				
Ocular pain intensity, average 1 week recall (0–10)	3.3 (2.7)	0.48	<0.0005	0.46	<0.0005
Burning (0–10)	2.7 (2.9)	0.41	<0.0005	0.43	<0.0005
Evoked Pain to Wind (0–10)	2.8 (3.0)	0.39	<0.0005	0.43	<0.0005
Evoked Pain by Light (0–10)	3.3 (3.2)	0.41	<0.0005	0.43	<0.0005
NPSI-Eye total score (0–100)	20.3 (20.7)	0.48	<0.0005	0.50	<0.0005
**Ocular surface measures**	**Mean (SD)**				
Osmolarity (mOsm/L)	305.0 (15.7)	−0.03	0.67	−0.02	0.83
Belmonte detection threshold (mL/min)	76.0 (42.4)	−0.17	0.02	−0.12	0.12
Belmonte pain threshold, (mL/min)	252.7 (126.3)	−0.08	0.27	−0.11	0.14
Ocular inflammation (InflammaDry) (0–3)	0.9 (0.9)	−0.16	0.02	−0.03	0.68
Tear Break Up Time (seconds)	8.9 (5.0)	−0.09	0.21	−0.05	0.47
Corneal Staining (0–15)	2.2 (2.4)	0.00	0.99	−0.02	0.81
Schrimer Score (mm wetting at 5 min)	12.0 (7.3)	−0.01	0.88	0.05	0.51
Eyelid Vascularity (0–3)	0.5 (0.7)	−0.05	0.05	−0.007	0.92
Meibum Quality (0–4)	2.0 (1.3)	−0.001	0.98	0.01	0.89
**Non-Ocular Indices**	**Mean (SD)**				
Non-ocular pain intensity, average 1 week recall (0–10)	4.9 (3.1)	0.54	<0.0005	0.60	<0.0005
Number of chronic pain conditions (0–21)	2.6 (1.5)	0.32	<0.0005	0.48	<0.0005
PTSD (PCL-M) (17–85)	40.8 (18.8)	0.40	<0.0005	0.52	<0.0005
Depression (PHQ9) (0–27)	9.7 (7.5)	0.52	<0.0005	0.55	<0.0005
Insomnia (ISI) (0–28)	13.5 (8.5)	0.38	<0.0005	0.47	<0.0005

SD-standard deviation; r-Pearson correlation coefficient; DEQ5-Dry Eye Questionnaire-5; OSDI-Ocular Surface Disease Index; NPSI-E-Neuropathic Pain Symptom Inventory modified—Eye; PCS-Pain Catastrophizing Scale; PHQ9-Patient health questionnaire 9; PTSD-Post traumatic Stress Disorder; PCL-M-PTSD Checklist, Military Version; ISI-Insomnia Severity Index.

**Table 2:** Model of Variables that Impact Pain Interference (MPI-Interference).

Associated Variables	β	SE	*p*-Value
Catastrophizing (PCS)	0.316	0.008	<0.0005
Non-ocular pain intensity, average 1 week recall	0.168	0.043	0.01
PTSD (PCL-M)	0.219	0.006	<0.0005
Number of pain conditions	0.188	0.071	0.001
Ethnicity	–0.112	0.228	0.02
OSDI	0.133	0.005	0.03

SE-standard error; PCS-Pain Catastrophizing Scale; PTSD-Post traumatic Stress Disorder; PCL-M-PTSD Checklist-Military version; OSDI-Ocular Surface Disease Index.
